# A case of spontaneous bilateral epidural hematoma associated with decreased coagulation factor XII activity: case report and literature review

**DOI:** 10.3389/fneur.2024.1460073

**Published:** 2024-09-23

**Authors:** Zhuxiao Tang, Ming Wang, Tao Xiong, Weixian Liu, Hu Sun, Jiangchun Ma

**Affiliations:** Brain Center, Zhejiang Hospital, Hangzhou, Zhejiang, China

**Keywords:** intracranial, spontaneous, non-traumatic, epidural hematoma, coagulation factor XII

## Abstract

Epidural hematoma typically manifests following craniocerebral trauma, stemming from injury to the meningeal artery or venous system, predominantly on one side. Instances of spontaneous epidural hematoma are uncommon, with occurrences of spontaneous bilateral epidural hematoma being exceedingly rare. Sickle cell disease, adjacent paranasal sinusitis, and tumor metastases are the most prevalent causes of spontaneous epidural hematoma. This case study presents an individual with abdominal liposarcoma exhibiting reduced coagulation factor XII activity, who experienced sudden unconsciousness due to spontaneous acute bilateral epidural hematoma, and subsequently achieved a favorable outcome following surgical intervention.

## Introduction

An intracranial acute epidural hematoma (AEDH) commonly arises in the space between the skull and the dura mater following a skull fracture, due to the rupture of the middle meningeal artery or venous system as a result of head trauma (1). Non-traumatic acute epidural hematoma, also referred to as spontaneous acute epidural hematoma, is a rare occurrence with less than 100 reported cases documented in current literature (1–5). Spontaneous epidural hematoma typically presents unilaterally, with bilateral occurrences being exceedingly rare, as evidenced by only approximately 13 reported cases (2, 4, 6–16) (Table 1). This condition is commonly linked to various underlying pathologies, such as adjacent infection, extradural metastasis, dural vascular malformations, and coagulation disorders (17). The present study details a unique case of spontaneous acute bilateral epidural hematoma following abdominal liposarcomatosis and prolonged exudate of digestive juices, characterized by decreased activity of coagulation factor XII. The patient experienced sudden loss of consciousness, but achieved favorable outcomes following surgical intervention.

**Table 1:** Reported cases of spontaneous bilateral epidural hematomas.

	Authors & Year	Age	Sex	Site of EDH	Cause of EDH
1	Kuwayama et al., 1985 (6)	21	Female	Temporal	Hypofibrinogenemia
2	Ishige et al., 1985 (7)	34	Male	Parietal & Occipital	SLE
3	Charles et al., 1987 (8)	34	Male	Occipital	Alcoholic brain atrophy
4	Grabel et al., 1989 (9)	2	Male	Unknown	Temporal
5	Dufour et al., 2001 (10)	36	Female	Parietal	Cavernous hemangioma
6	Melike et al., 2004 (11)	9	Male	Parietal	Eosinophilic granuloma of skull
7	Ng et al., 2004 (12)	23	Female	Frontal	Unknown
8	Verma et al., 2007 (13)	3	Male	Frontal	Scurvy
9	Li et al., 2012 (14)	14	Female	Temporal & parietal	Low intracranial pressure
10	Hettige et al., 2015 (15)	7	Female	Temporal	Sickle cell disease
11	Khursheed et al., 2017 (16)	40	Male	Frontal	Chronic kidney disease
12	Lin et al., 2023 (4)	21	Male	Frontal	Chronic sinusitis
13	Aljohani et al., 2024 (2)	10	Female	Parietal	Sickle cell disease

## Case presentation

**Figure 1:**
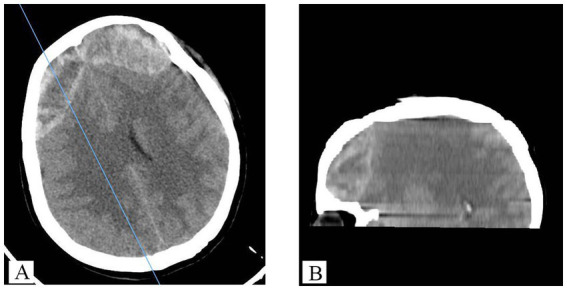
Preoperative intracranial epidural hematoma is shown from different sections: **(A)** cross-section, **(B)** sagittal section.

**Figure 2:**
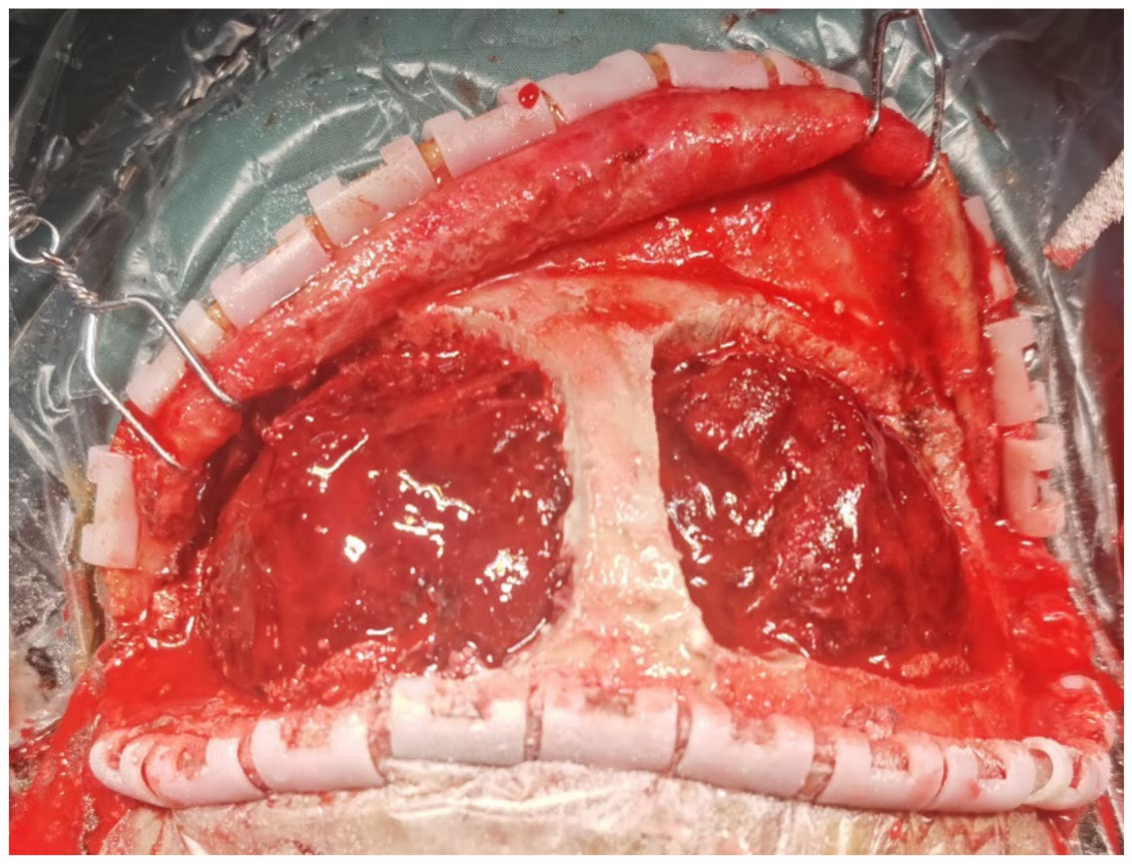
Intraoperatively, after opening the bilateral frontal bone flaps adjacent to the sagittal sinus, a large amount of epidural hematoma is observed.

A 45-year-old female patient was admitted to the local hospital 5 years ago due to persistent weight loss. Upon examination, extensive thickening of the abdominal colon, small intestine, mesentery, and greater omentum was observed, leading to the performance of surgical intervention. Postoperative pathology revealed a diagnosis of highly differentiated liposarcoma, prompting the initiation of adjuvant therapy. However, over 1 month following the operation, the patient experienced anastomotic abdominal wall leakage, resulting in the leakage of digestive juices through the skin. Despite repeated surgical repairs, there was no improvement in the patient’s condition. The patient was readmitted to the anorectal surgery department of our hospital due to an escalation of intestinal leakage. While hospitalized, the patient experienced sudden and persistent frontal pain in the evening, followed by unconsciousness and bilateral pupil dilation, with a Glasgow Coma Scale score of 7 (E1 + V1 + M5). An urgent head CT scan revealed a “double frontal epidural hematoma” (Figure 1), prompting emergency “craniotomy hematoma removal.” The patient had no history of trauma, nor did she have any family history of genetic disorders. Additionally, she had not taken anticoagulant drugs or oral contraceptives. During the surgical procedure, no apparent scalp or skull trauma was observed. Upon opening the skull, a significant epidural hematoma was discovered on both frontal lobes (Figure 2). There were no indications of dural vascular abnormalities or tumor metastasis, and no abnormal blood coagulation was noted on the day of the operation. Subsequent analysis of coagulation factors post-operation revealed a decrease in the activity of multiple factors. However, in subsequent follow-up examinations, it was observed that the activity of the other factors had returned to normal levels exception of factor XII (Table 2). The antinuclear antibodies, autoimmune disease-related antibodies, and thromboelastogram all exhibited normal results, except for a minor elevation in anti-Ro-52 antibody levels.

**Table 2:** The results of coagulation factor activity assessed on the day of surgery and during two subsequent postoperative follow-up visits.

Coagulation factor activity	2023-11-24	2023-12-01	2023-12-15
II (Factor II)	61.3% (Low)	84.8% (Normal)	88.4% (Normal)
V (Factor V)	79.3% (Normal)	126.3% (High)	98.7% (Normal)
VII (Factor VII)	84.3% (Normal)	72.7% (Normal)	87.8% (Normal)
IX (Factor IX)	61.7% (Low)	77.3% (Normal)	83.1% (Normal)
X (Factor X)	69.9% (Low)	84.2% (Normal)	89.4% (Normal)
XI (Factor XI)	43.7% (Low)	69.8% (Low)	88.9% (Normal)
XII (Factor XII)	34.3% (Low)	36.1% (Low)	38.6% (Low)

**Figure 3:**
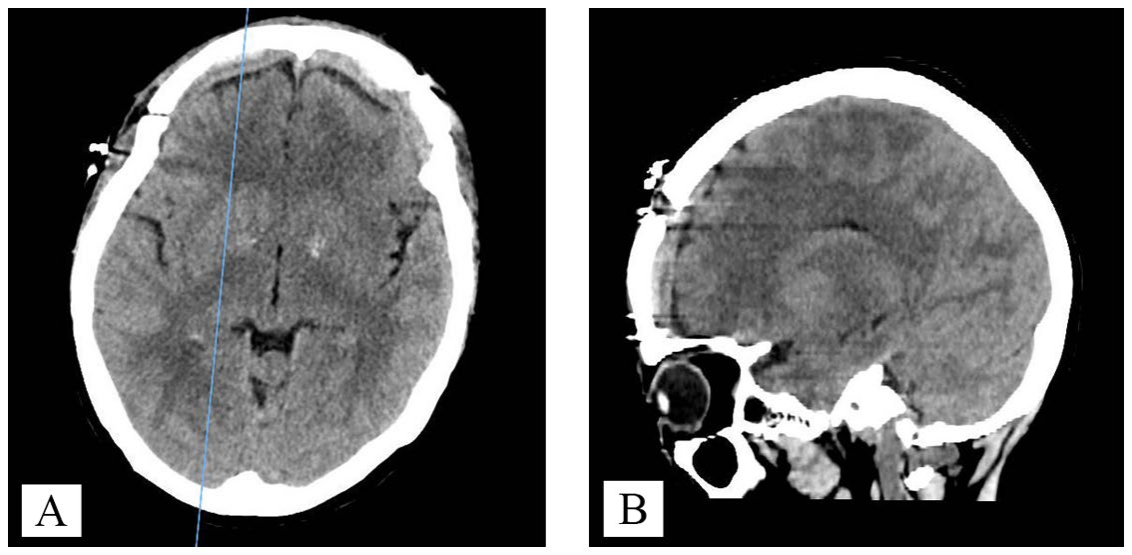
Postoperatively, a cranial CT scan was performed: **(A)** cross-section, **(B)** sagittal section.

Following the surgical procedure, the patients exhibited restored consciousness, satisfactory limb movement, and absence of neurological deficits. Postoperative CT imaging confirmed successful clearance of the hematoma (Figure 3). At the six-month follow-up, the patients continued to recover without any recurrence of acute epidural hematoma.

## Discussion

Intracranial epidural hematoma is a relatively rare yet grave consequence of head trauma, often presenting concurrently with skull fracture and suture diastasis. Although its precise prevalence remains undetermined, estimates suggest it comprises 1–4% of traumatic head injury cases and 5–15% of postmortem examinations (17). Spontaneous occurrences of epidural hematoma are infrequent but constitute a particularly uncommon neurological emergency necessitating prompt evaluation and intervention (18).

In a comprehensive analysis of 77 cases of intracranial spontaneous acute epidural hematoma, Sickle cell disease (SCD), adjacent sinusitis, and metastatic hepatocellular carcinoma (HCC) were identified as the predominant etiologies, accounting for 32.5, 22.1, and 19.5% of cases, respectively (1). The suggested pathophysiological mechanisms underlying acute epidural hematoma in the context of SCD involve bone infarction, microfractures resulting from hematopoietic dilatation, and blood leakage due to the accumulation of sickle cells within the plate barrier vein (2). The presence of diffuse inflammation in the paranasal sinuses can lead to the infiltration of air into the epidural space from the sinuses, causing the separation of the dura mater from the skull. Sinusitis can progress to involve the meningeal artery wall through the blood vessels in the cranial plate barrier, resulting in hemorrhage from a weakened meningeal artery entering the epidural space (4). Cases of acute epidural hematoma (AEDH) have been reported in patients with metastatic tumors in the skull, such as those originating from lung, ovarian, esophageal, Ewing’s sarcoma, and Langerhans cell histiocytosis. While metastasis to the skull is a common occurrence in patients with lung, breast, and prostate cancer, it is relatively rare in patients with hepatocellular carcinoma (HCC) (8). However, cases of spontaneous acute epidural hematoma caused by tumor metastasis are predominantly associated with metastatic foci of HCC, a recognized risk factor for bleeding. When cancer cells metastasize to the dura mater or skull instead of the brain parenchyma, metastatic bleeding can extend to the epidural space (3).

Among the cohort of 14 patients diagnosed with bilateral spontaneous epidural hematoma, including the case under consideration, the etiology varied, with a higher prevalence in younger individuals and a lack of specificity in the hematoma’s location.

The patient under consideration in this case study presents with abdominal liposarcoma of high differentiation. While tumor metastasis can potentially result in epidural hematoma, there have been no documented instances of epidural hemorrhage stemming from liposarcoma metastasis. Furthermore, both the patient’s CT scans and intraoperative observations do not indicate evidence of tumor metastasis. The patient has a prolonged history of intestinal fistula following a previous abdominal liposarcoma operation; however, pre-and post-operative coagulation function tests reveal no significant abnormalities in PT, APTT, INR, or other relevant parameters. The sole anomaly observed in this patient was a reduction in coagulation factor XII activity. Acquired FXII deficiency is linked to diminished synthesis (as seen in liver disease), heightened loss (as seen in nephrotic syndrome), and excessive consumption (as seen in endotoxin-induced septicemia or disseminated intravascular coagulation), along with other coagulation irregularities. Instances of specific FXII inhibitors are uncommon. Additionally, FXII is spontaneously converted to FXIIa upon interaction with a negatively charged surface, contributing to the physiological mechanism of blood coagulation and serving a crucial role in anti-thrombotic and promoting fibrinolysis processes. Due to the crucial involvement of FXII in the endogenous coagulation pathway, its deficiency results in a marked prolongation of APTT. FXII deficiency typically does not predispose individuals to spontaneous bleeding, and there have been no reported instances of intracranial hemorrhage associated with this condition. Following a six-month follow-up period, the patient exhibited satisfactory recovery, apart from an abdominal intestinal fistula issue. Despite this, there was no recurrence of spontaneous intracranial hemorrhage. Consequently, the etiology of acute spontaneous epidural hematoma in this patient remains unclear.

## Conclusion

While the occurrence of spontaneous acute intracranial epidural hematoma is rare, the rapid progression of the clinical course is attributed to heightened intracranial pressure and brain compression resulting from the hematoma. Timely craniotomy can lead to complete recovery from the hematoma. However, further investigation is required to identify the underlying cause of spontaneous bleeding and to implement appropriate treatment to prevent disease recurrence. Our medical records suggest that XII coagulation factor may play a role in spontaneous intracranial hemorrhage, which is worthy of our further study.

## Data Availability

The original contributions presented in the study are included in the article/supplementary material, further inquiries can be directed to the corresponding author.
